# Acculturation and glycaemic control in Arab immigrants with type 2 diabetes in Australia

**DOI:** 10.1007/s00125-023-06081-5

**Published:** 2024-01-12

**Authors:** Hamzah Alzubaidi, Vitor H. Oliveira, Catarina Samorinha, Kevin Mc Namara, Jonathan E. Shaw

**Affiliations:** 1 Pharmacy Practice and Pharmacotherapeutics, College of Pharmacy, University of Sharjah, Sharjah, United Arab Emirates; 2 Research Institute for Medical and Health Sciences, University of Sharjah, Sharjah, United Arab Emirates; 3 School of Medicine, Deakin Rural Health, Deakin University Faculty of Health, Warrnambool, VIC Australia; 4 inED Centre for Research and Innovation in Education, School of Education, Polytechnic of Porto, Porto, Portugal; 5 Clinical Diabetes and Epidemiology, Baker Heart and Diabetes Institute, Melbourne, VIC Australia; 6 Department of Epidemiology and Preventive Medicine, Monash University, Melbourne, VIC Australia

**Keywords:** Acculturation, Glycated haemoglobin A_1c_, Type 2 diabetes

## Abstract

**Aims/hypothesis:**

This study aimed to investigate acculturation’s direct and mediated effects on HbA_1c_ levels in individuals with type 2 diabetes from Arabic-speaking countries that are members of the Arab League who have emigrated to Australia.

**Methods:**

In this multicentre cross-sectional study, we recruited 382 Arabic-speaking immigrants who were born in any of the 22 countries of the Arab League and who had type 2 diabetes from different healthcare settings in Australia. HbA_1c_ levels were retrieved from medical records. A validated self-report questionnaire was used to assess behavioural and psychosocial outcomes. Acculturation was measured using the General Acculturation Index and the Adherence to Traditional Values tool. We used structural equation modelling to test mediation hypotheses.

**Results:**

Participants had a mean HbA_1c_ value of 63.9 mmol/mol (8.0%), a low acculturation level (mean±SD: 1.9±0.6; range: 1–5) and highly adhered to traditional values (mean General Acculturation Index value: 3.7±0.7; range: 1–5). Higher HbA_1c_ was associated with lower acculturation levels (Pearson correlation coefficient [*r*] = −0.32, *p*<0.01) and higher adherence to traditional values (*r*=0.35, *p*<0.01). Self-efficacy, health literacy and self-care activities partially mediated the relationship between acculturation and HbA_1c_.

**Conclusions/interpretation:**

Among Arab immigrants in Australia with type 2 diabetes, the degree of acculturation is related to glycaemic control, suggesting possible avenues for new interventions.

**Graphical Abstract:**

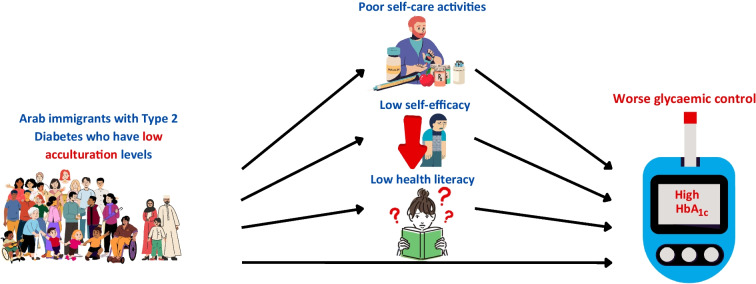

**Supplementary Information:**

The online version contains peer-reviewed but unedited supplementary material available at 10.1007/s00125-023-06081-5.


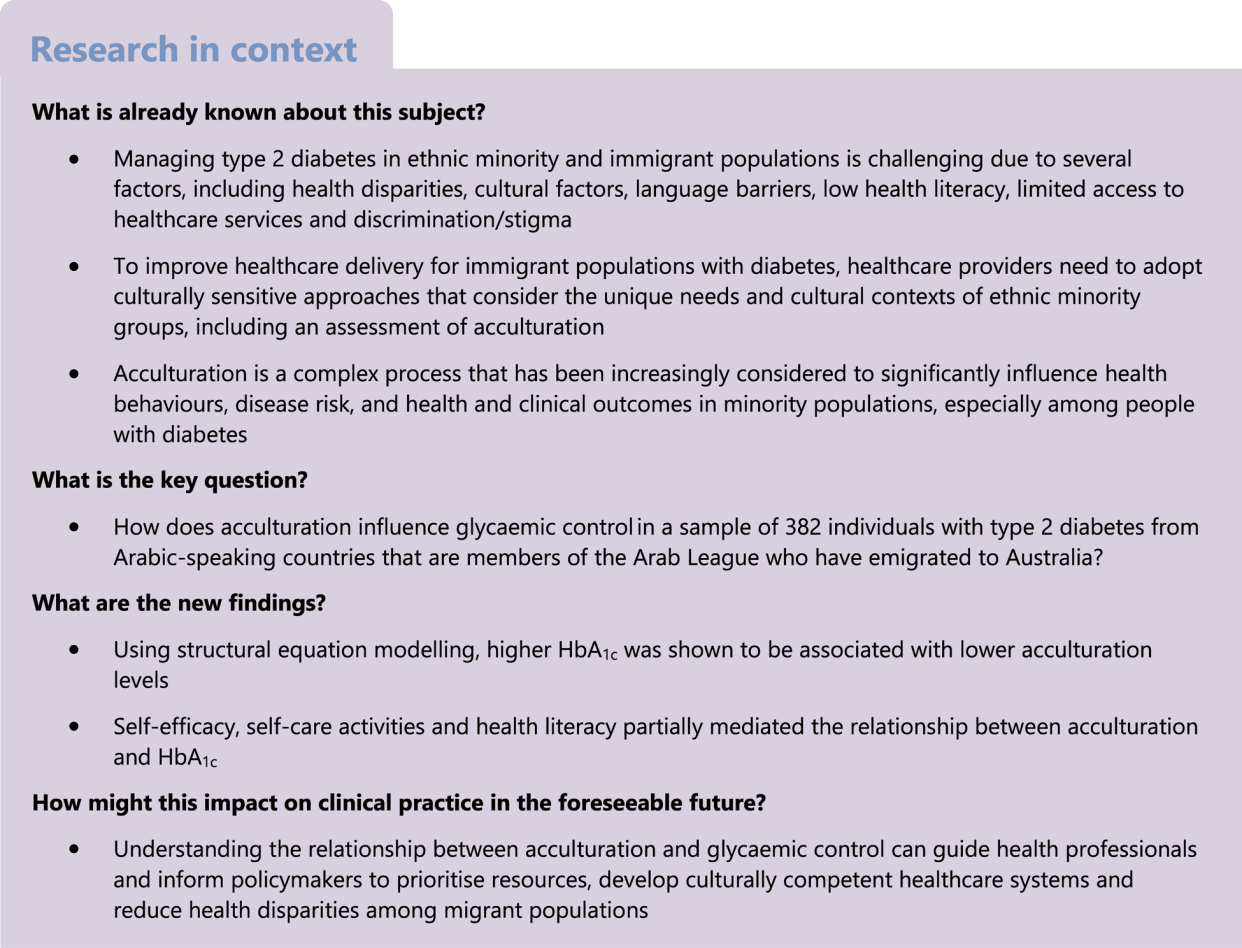


## Introduction

Type 2 diabetes is more challenging among minority populations due to disparities in healthcare access and higher morbidity and mortality rates than among non-minority individuals [1, 2]. Addressing diabetes challenges among these vulnerable populations requires multi-faceted, culturally appropriate interventions [3]. However, intervention development requires a comprehensive understanding of behavioural determinants in the target group. Arab immigrants (individuals who have emigrated from Arabic-speaking countries that are members of the Arab League) are among the least studied ethnic groups in this regard [4], despite higher type 2 diabetes prevalence, worse self-care practices, worse adherence to prescribed medications and poorer glycaemic control in these individuals than in the general population [5–7], highlighting the need for appropriate intervention.

Acculturation, the cultural and psychological change process resulting from continuous first-hand contact between people of different cultures [8, 9], is increasingly considered to significantly influence health behaviours, disease risk and health and clinical outcomes in minority populations [10, 11]. There are evidence gaps globally regarding the impact of acculturation on glycaemic control (HbA_1c_ levels), including among Arab migrants. However, self-efficacy, illness perception, self-care activities (physical activities, dietary behaviours, self-monitoring of blood glucose and foot care) and health literacy have been associated with diabetes self-management behaviour and clinical outcomes among Arab immigrants. We postulated that they may mediate the relationship between acculturation and HbA_1c_ levels [7, 12, 13]. Our study investigated the direct and mediated effects of acculturation on HbA_1c_ levels in Arab immigrants in Australia using structural equation modelling.

## Methods

This multicentre cross-sectional study was conducted in various health settings (diabetes outpatient clinics at three major hospitals, general medical practices and community support groups) in metropolitan and rural areas of Victoria, Australia.

### Participants

Arabic-speaking first-generation immigrants with type 2 diabetes, whose first language was Arabic and who were born in any of the 22 countries of the Arab League, completed a validated self-report paper-based questionnaire (see below for details). Medical records for these individuals were also accessed.

### Instrument and variables

The validated self-report paper-based questionnaire was purposefully designed for a larger research project examining diabetes care among the Arabic-speaking immigrant population in Australia. Besides acculturation, it assessed sociodemographic and health characteristics, behavioural and psychosocial factors, and clinical outcomes. Sex of the participants was self-reported.

The acculturation level of Arabic-speaking immigrants was used as the independent variable, operationalised as the degree to which they have integrated into Australian society. The acculturation level was assessed using two measures, the first being an adaptation of the General Acculturation Index [10], composed of three questions regarding preferred language for reading and speaking, current circle of friends and sense of pride about having an Arabic background (electronic supplementary material [ESM] Table 1). The arithmetic mean of the total score was calculated (possible range: 1–5). The second tool assessed adherence to traditional values and attitudes, which was developed by Jaber et al [4] specifically for Arab immigrants. It contains five items, and responses range from 1 (low adherence to traditional values) to 5 (high adherence to traditional values) (ESM Table 1). A total score was calculated as the arithmetic mean of all items.

Functional health literacy was measured using three validated items assessing difficulties in reading medical forms or learning about medical conditions, and confidence in filling medical forms [14]. Participants reported their responses on a five-point Likert-type scale and total score was computed. Diabetes illness beliefs were assessed using the validated Brief Illness Perception Questionnaire (BIPQ), which has nine items measuring cognitive illness representation, affective responses to illness and overall understanding of the disease [15], and the arithmetic mean was calculated. Higher scores suggested that participants had more negative perceptions about their illness. Participants' adherence to beneficial dietary behaviours, physical activity, blood glucose monitoring and foot care was assessed using the Summary of Diabetes Self-Care Activities measure [16]. The data are reported as the arithmetic mean number of days that self-care activities were performed during the previous week. Self-efficacy was measured by the reported confidence in conducting two tasks: taking diabetes medicines as prescribed by the treating physician and carrying out self-care activities successfully [17]; participants recorded their responses to both items on a five-point Likert scale and the arithmetic mean was calculated for analysis. Health status was assessed by a self-report item with six categories (ranging from ‘very bad’ to ‘excellent’), adapted from the Organisation for Economic Co-operation and Development Health Statistics questionnaire [18]. For the analysis, a total score was used, in which a higher score reflected better perceived health status. The latest HbA_1c_ levels for each participant was obtained with participant consent from medical records (within the preceding 12 months).

Ethical approvals were obtained from the Monash University Human Research Ethics Committee (CF09/0956: 2009000462) and the human ethics committees of all participating hospitals.

#### Sample size

The sample size was determined based on a minimum correlation (*r*) strength of 0.20, an *α*-level of 5% and a power of 80%. It was estimated that a minimum of 180 Arabic-speaking immigrants were needed to complete the survey.

### Data analysis

**Fig. 1:**
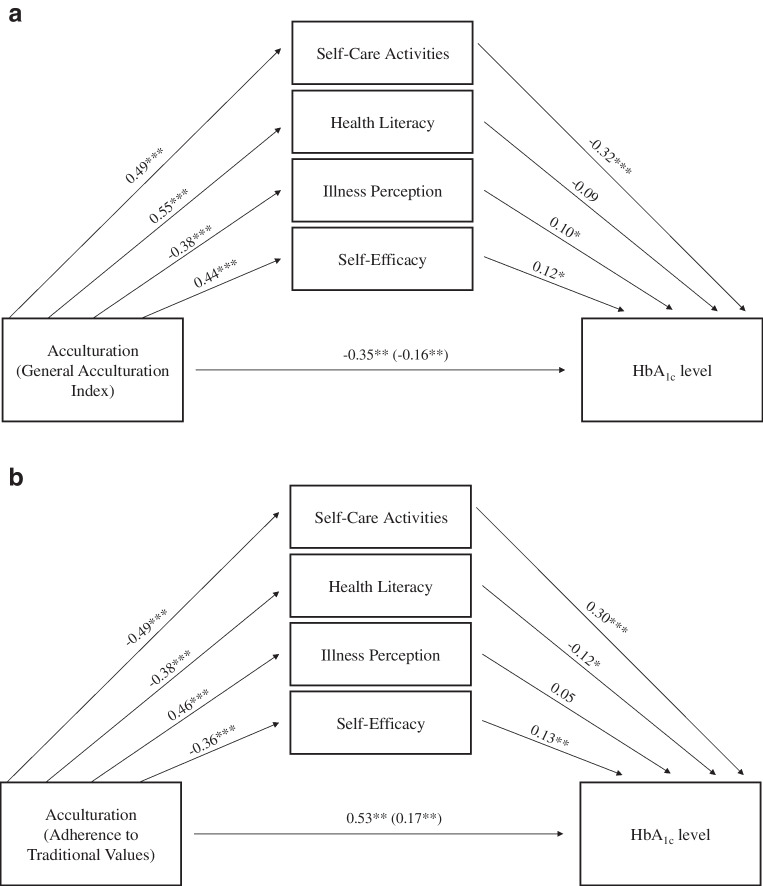
Mediation path diagrams illustrating the direct and indirect impact of acculturation on HbA_1c_ level, with standardised regression coefficients (*β*), using (**a**) the General Acculturation Index as a predictor (Model 1) or (**b**) adherence to traditional values as a predictor (Model 2). Standardised regression coefficient values are shown in brackets and represent the partially mediated change in the relationship between the predictor and the outcome when controlling for the effect of mediators and the control variable. *p<0.05, **p<0.01, ***p<0.001

Variables of interest were analysed for distribution normality and no transformation was considered necessary. A descriptive analysis of the main variables of interest was performed. Categorical variables are presented as absolute frequencies (*n*) and percentages (%), and continuous variables as mean±SD. A set of Pearson correlation coefficients (*r*) was calculated to test the relationships between acculturation and all sociodemographic and health characteristics (data not shown). Only the variables with statistically significant correlations were entered in the models. Structural equation modelling using SPSS Amos (Version 27 [2006]; Chicago, IL, USA; supplied by Universidade do Minho, Braga, Portugal: https://gabinetetecnico.dps.uminho.pt/index.php/instalacao-de-software/) was used to test the mediation hypothesis regarding the effect of acculturation on HbA1_c_ levels in two models (in Model 1, the General Acculturation Index was used as a predictor, while in Model 2, adherence to traditional values was used as a predictor) through self-efficacy (‘indirect path 1’), illness perception (‘indirect path 2’), health literacy (‘indirect path 3’) and self-care activities (‘indirect path 4’), while controlling for health status (confounder). Maximum likelihood estimation was used to calculate total, direct and indirect effects, and bias-corrected accelerated 95% CI values (using 1000 bootstrap resamples) were calculated. Three estimands were defined to obtain specific path estimates representing each indirect path. Standardised regression coefficients and the explained variance are reported. Mediation path diagrams illustrating the direct and indirect impact of acculturation on HbA_1c_ level are presented (with unadjusted standardised regression coefficients; Fig. 1). In the case of missing values, data imputation was used with the regression imputation method (missing data for most variables was <2%; see ESM Table 2).

## Results

This study included 382 Arabic-speaking immigrants with type 2 diabetes (participation rate=93.2%). Half of the participants were male (*n*=191; 50.0%) and the mean age was 57.9±8.0 years (ESM Table 2). Most were married (*n*=261; 68.3%) and had at least a high-school level of education (*n*=289; 75.7%). Most participants were not working, including homemakers and unemployed or retired individuals (*n*=228; 59.7%). Most immigrants were born in Lebanon (*n*=124; 32.5%), Egypt (*n*=95; 24.9%) or Iraq (*n*=70; 18.3%) and had lived in Australia for 19.1±8.3 years. Participants had diagnosed diabetes for 7.1±4.7 years and reported a perceived health status of 3.3±1.1 (range: 1–6). Participants had a mean HbA_1c_ value of 63.9 mmol/mol (8.0%) (range:4.0–13.1%) and 87.3% had HbA_1c_ values above 53.0 mmol/mol (7.0%).

Overall, participants had a mean functional health literacy of 2.46±0.97; range: 1–5), mean illness perception score of 5.54±0.73; range: 0–10), and a high mean self-efficacy score regarding taking diabetes medications and self-care activities (3.60±0.61; range: 1–5). Participants were engaged in self-care activities on 2.78±1.39 days per week. When analysing the acculturation levels in our sample, participants presented an overall low acculturation level (mean General Acculturation Index value: 1.9±0.6; range: 1–5) and high adherence to traditional values (mean: 3.7±0.7; range: 1–5).

All variables of interest significantly correlated with each other at the significance level of *p*<0.05 and were subsequently included in mediation Model 1 and 2 (ESM Table 3). No differences in HbA_1c_ were found according to the sex of participants (data not shown).

Higher HbA_1c_ was associated with lower acculturation levels, as measured by the General Acculturation Index (Pearson correlation coefficient = −0.32; *p*<0.01), and with higher adherence to traditional values (i.e. lower acculturation levels), as measured by the Adherence to Traditional Values tool (*r*=0.35; *p*<0.01). Therefore, a mediation analysis was conducted with these variables to examine the mediating effect of self-efficacy, illness perception, health literacy and self-care activities in the relationship between acculturation and HbA_1c_. Model 1, which used the General Acculturation Index as a predictor, explained 29% of the variance in HbA_1c_, with the total, direct and indirect effects being significant, indicating both direct and indirect (mediated) effects of acculturation on HbA_1c_ (Table 1, Fig. 1a). Importantly, acculturation remained a significant predictor of HbA_1c_ even after controlling for the effects of the mediators alongside the control variable (health status) (*β*=−0.190, *p*=0.002) (Table 1). Specifically, in this model, self-efficacy and diabetes self-care activities partially mediated the relationship between acculturation and HbA_1c_, while illness perception and health literacy were not statistically significant mediators. In Model 2, which used adherence to traditional values as a predictor, the total, direct and indirect effects of the variables on the relationship between acculturation and HbA_1c_ were statistically significant, with the total effect explaining 27% of the variance in HbA_1c_ (Table 1, Fig. 1b). Acculturation was retained as a significant predictor of HbA_1c_ after controlling for the effects of the mediating variables alongside health status (*β*=−0.17, *p*=0.003) (Table 1). Specifically, indirect paths 1, 3 and 4, relating to the effects of self-efficacy, health literacy and self-care activities, were independently and statistically significant in this model, suggesting that these variables partially mediated the relationship between acculturation and glycaemic control. Illness perception was not a statistically significant mediator.

**Table 1:** Mediation results for the effect of acculturation on HbA_1c_ by self-efficacy, illness perception, health literacy and self-care activities, controlling for health status

Model	Estimate (*β*)^a^	SE	*p* value	BCa 95% CI (lower, upper)	*R*^2 b^
Model 1^c^					
Total effects: Acc (GAI)* →* HbA_1c_	−0.352	0.058	0.002	−0.47, −0.24	0.29
Direct effects: Acc (GAI) → HbA_1c_	−0.162	0.060	0.010	−0.27, −0.04	
Indirect effects	−0.190	0.049	0.002	−0.29, −0.10	
Indirect path 1: Acc → Self-Ef → HbA_1c_	0.103	0.037	0.005	0.04, 0.19	
Indirect path 2: Acc → Ill-Perc → HbA_1c_	−0.072	0.043	0.118	−0.16, 0.02	
Indirect path 3: Acc → Hth-Lit → HbA_1c_	−0.095	0.052	0.064	−0.21, 0.01	
Indirect path 4: Acc → Self-Care → HbA_1c_	−0.298	0.063	0.001	−0.44, −0.19	
Model 2^d^					
Total effects: Acc (ATV) → HbA_1c_	0.530	0.080	0.002	0.37, 0.68	0.27
Direct effects: Acc (ATV) → HbA_1c_	0.170	0.055	0.003	0.06, 0.28	
Indirect effects	0.167	0.045	0.003	0.08, 0.25	
Indirect path 1: Acc → Self-Ef → HbA_1c_	−0.073	0.024	0.001	−0.13, −0.03	
Indirect path 2: Acc → Ill-Perc → HbA_1c_	0.036	0.043	0.399	−0.05, 0.12	
Indirect path 3: Acc → Hth-Lit → HbA_1c_	0.070	0.030	0.009	0.02, 0.14	
Indirect path 4: Acc → Self-Care → HbA_1c_	0.230	0.054	0.002	0.13, 0.35	

Displayed are mediation results for the effect of acculturation on HbA_1c_ by self-efficacy (‘indirect path 1’), illness perception (‘indirect path 2’), health literacy (‘indirect path 3’) and self-care activities (‘indirect path 4’), controlling for health status

^a^Estimates are standardised regression coefficients

^b^*R*^2^ represents the squared multiple correlations, i.e. the percentage of variance explained by the model

^c^For Model 1, the General Acculturation Index was used as a predictor and health status was used as the control variable (for Model 1, health status *→* HbA_1c_: *β*=−0.19, *p*=0.002)

^d^For Model 2, adherence to traditional values was used as a predictor and health status was used as the control variable (for Model 2, health status *→* HbA_1c_: *β*=−0.17, *p*=0.003)

Acc, acculturation; ATV, Adherence to Traditional Values; BCa, bias-corrected accelerated; GAI, General Acculturation Index; Hth-Lit, health literacy; Ill-Perc, illness perception; Self-Care, self-care activities; Self-Ef, self-efficacy

## Discussion

This study has shed light on the relevance of acculturation in glycaemic control in Arab immigrants with type 2 diabetes. Using two measures of acculturation, data consistently showed that immigrants with lower acculturation levels had worse HbA_1c_, regardless of the sex of the participants. Furthermore, it highlighted the mediating role of health literacy, self-efficacy and self-care activities on glycaemic control. Recognising that acculturation influences self-efficacy, health literacy and self-care activities (e.g., dietary choices and physical activity), which, in turn, influence glycaemic control [12–14], clinicians should consider these factors when treating individuals with type 2 diabetes. These results highlight the importance of assessing acculturation as this measure allows healthcare professionals to understand and examine the cultural identities, experiences and perspectives related to diabetes management, thus improving healthcare delivery [7]. Additionally, such assessments could inform the design of culturally appropriate diabetes healthcare services.

Using a robust statistical technique that accounts for measurement error (structural equation modelling) to test the complex relationships between the variables that we analysed guarantees high accuracy of the estimates provided. In addition, it allowed us to gain insight into the mechanisms underlying the associations that we observed. To address the challenges of performing mediation analysis using observational data, we assessed the statistical significance of the indirect effects. These effects were estimated using CIs and were adjusted for the confounding variable of health status. This was done to ensure that the effect estimates could be interpreted causally. Future studies could clarify the causal structure of the mediation model by reporting directed acyclic graphs. We recognise that this study did not account for all factors that may have mediated the relationship between acculturation and HbA_1c_, such as social discrimination and access to care. In addition, the representativeness of the study sample to the larger population of interest could not be ascertained. Moreover, future studies should examine acculturation longitudinally to establish causality and understand the direction of the associations, such as the relationship between glycaemic control and illness perception, in the broader context of individual and environmental factors affecting people with diabetes.

### Supplementary Information

Below is the link to the electronic supplementary material.

Supplementary file1 (PDF 156 KB)

## Data Availability

Data are available on request from the authors.
