# Supplementary material for: Acculturation and glycaemic control in Arab immigrants with type 2 diabetes in Australia
Source: Diabetologia. 2024 Jan 12;67(4):663–9. doi: 10.1007/s00125-023-06081-5 (PMC10904404; doi:10.1007/s00125-023-06081-5)
Supplement: Supplementary file 1 — Supplementary file1 (PDF 156 KB) [file 125_2023_6081_MOESM1_ESM.pdf]

Electronic Supplementary Material (ESM)

ESM Table 1 – Items and response categories of the tools used to assess acculturation

Tool	Items	Response categories
General Acculturation Index (adapted from Harmon et al., 1996)[1]	1. I read/speak:	1. Only Arabic, 2. Arabic better than English, 3. Both equally well, 4. English better than Arabic, 5. Only English
	2. Currently my circle of friends are:	1. Only Arabic, 2. Mainly Arabic, 3. Both Arabic and Australian, 4. Mainly Australian, 5. Only Australian
	3. In relation to having an Arabic-background, I feel:	1. Very proud, 2. Proud, 3. Somewhat proud, 4. Little pride, 5. No pride
Adherence to traditional values and attitudes (Jaber et al., 2003)[2]	1. Duty to one's family comes before personal desires.	1. Strongly disagree, 2. Disagree, 3. Neither agree nor disagree, 4. Agree, 5. Strongly agree
	2. Australian of Arabic-background shouldn't disagree among themselves if there are Caucasians around	1. Strongly disagree, 2. Disagree, 3. Neither agree nor disagree, 4. Agree, 5. Strongly agree
	3. It would be more comfortable to live within an Arabic-speaking community in Australia.	1. Strongly disagree, 2. Disagree, 3. Neither agree nor disagree, 4. Agree, 5. Strongly agree
	4. In the Arabic-speaking community, human relationships are more warm and comfortable than the outside society	1. Strongly disagree, 2. Disagree, 3. Neither agree nor disagree, 4. Agree, 5. Strongly agree
	5. Marrying within the Arabic-speaking community is good	1. Strongly disagree, 2. Disagree, 3. Neither agree nor disagree, 4. Agree, 5. Strongly agree

[1]Harmon MP, Castro FG, Coe K. Acculturation and cervical cancer: Knowledge, beliefs, and behaviors of Hispanic women. Women and Health. 1996;24(3):37-57. doi:10.1300/J013v24n03_03.

[2]Jaber LA, Brown MB, Hammad A, Zhu Q, Herman WH. Lack of Acculturation Is a Risk Factor for Diabetes in Arab Immigrants in the U.S. *Diabetes Care*. 2003;26(7):2010-2014. doi:10.2337/diacare.26.7.2010.

ESM Table 2 - Participant sociodemographic and clinical characteristics (N= 382)

**Characteristics**	
Gender, *n* (%)	
Male	191 (50.0)
Female	191 (50.0)
Age (years), mean±SD	57.9±8.0
Educational level, *n* (%)	
≤ High school	289 (75.7)
> High school	88 (23.0)
Missing	5 (1.3)
Employment status, *n* (%)	
Employed	149 (39.0)
Not employed	228 (59.7)
Missing	5 (1.3)
Marital status, *n* (%)	
Married	261 (68.3)
Not married	112 (29.3)
Missing	9 (2.4)
Country of origin, *n* (%)	
Egypt	95 (24.9)
Iraq	70 (18.3)
Lebanon	124 (32.5)
Other[a]	93 (24.3)
Length of stay in Australia (years), mean±SD	19.1±8.3
Diabetes co-morbidities, *n* (%)	
Yes	311 (81.4)
No	70 (18.3)
Missing	1 (0.3)
Insulin use, *n* (%)	
Yes	99 (25.9)
No	253 (66.2)
Missing	30 (7.9)
Diabetes duration (years), mean±SD	7.1±4.7
Health status, mean±SD	3.3±1.1
HbA1c, mmol/mol	63.9±6.1
HbA1c, %±SD	8.0±1.2

[a]Other countries include: Jordan (n=12), Kuwait (n=5), Palestine (n=7), Somalia (n=45), Sudan (n=8), Syria (n=13) and United Arab Emirates (n=3); SD= Standard Deviation.

ESM Table 3 - Correlation coefficients between the study's variables

	1	2	3	4	5	6	7	8
1. General Acculturation Index	–	-0.59**	-0.32**	0.44**	-0.37**	0.55**	0.41**	0.49**
2. Adherence to Traditional Values		–	0.35**	-0.35**	0.45**	-0.37**	-0.26**	-0.49**
3. Glycaemic Control			–	-0.13*	0.26**	-0.25**	-0.30**	-0.38**
4. Self-Efficacy				–	-0.47**	0.24**	0.30**	0.60**
5. Beliefs about Illness					–	-0.10*	-0.43**	-0.65**
6. Health Literacy						–	0.28**	0.22**
7. Health Status							–	0.48**
8. Self-Care Activities								–

Note. *p < 0.05, **p < 0.01.
